# A multi-criteria assessment of Japanese Kanji learning difficulty for educational prioritization using ANP–VIKOR

**DOI:** 10.3389/fpsyg.2026.1902499

**Published:** 2026-08-10

**Authors:** Lin Liao, Nobuharu Sato

**Affiliations:** 1 School of Foreign Studies, Hunan University of Science and Technology, Xiangtan, Hunan, China; 2 Graduate School of Humanities and Social Sciences, Hiroshima University, Hiroshima, Japan

**Keywords:** analytic network process, educational decision support, Japanese Kanji learning, Kanji learning difficulty, VIKOR

## Abstract

**Introduction:**

Japanese Kanji acquisition remains a major challenge for non-native learners because character learning involves multiple cognitive and linguistic factors, including visual complexity, semantic transparency, usage frequency, and pronunciation variability. Although previous studies have examined individual aspects of Kanji difficulty, few have integrated these factors within a transparent multi-criteria decision-making framework.

**Methods:**

This study proposes a hybrid Analytic Network Process (ANP) and VlseKriterijumska Optimizacija I Kompromisno Resenje (VIKOR) framework to evaluate the difficulty of learning Kanji. Four evaluation criteria, namely stroke count, frequency of use, semantic transparency, and phonetic variability, were identified through a literature review and validated by an expert panel. ANP was used to determine the relative importance of the criteria, while VIKOR generated compromise rankings for representative high-frequency Kanji characters. Learner ratings from 50 non-native Japanese learners and expert evaluations were incorporated into the decision-making process.

**Results:**

The ANP analysis identified stroke count as the most influential criterion (0.30), followed by frequency of use (0.25), semantic transparency (0.25), and phonetic variability (0.20). The VIKOR analysis ranked **人** as the least difficult Kanji and **学** as the most difficult among the evaluated characters. Sensitivity analysis demonstrated stable rankings under different decision-making scenarios, indicating the methodological robustness of the proposed framework.

**Conclusion:**

The proposed ANP–VIKOR framework provides a transparent and systematic approach for evaluating Kanji learning difficulty by integrating expert knowledge, learner perceptions, and multiple linguistic criteria. The resulting rankings provide a transparent methodological framework for Kanji prioritization and educational planning. The proposed framework serves as a decision-support tool and provides a foundation for future classroom-based validation and educational research.

## 1 Introduction

Japanese Kanji characters are among the most visually and cognitively demanding components of the Japanese writing system (Inoue et al., 2022; Otsuka and Murai, 2021). Each Kanji encodes complex layers of meaning, combining pictographic elements, multiple pronunciations (on’yomi and kun’yomi), and contextual usage patterns that have evolved over centuries (Rose, 2017; Van et al., 2024). For learners, particularly non-native speakers, mastering Kanji involves not only memorizing a large set of characters but also navigating their varying degrees of semantic transparency, phonetic irregularities, and frequency of use in daily communication (Li et al., 2021; Shao et al., 2025). Given this complexity, prioritizing Kanji for educational purposes has been a longstanding challenge in language pedagogy (Konoeda, 2024; Koyama, 2022).

Traditional approaches to Kanji instruction have often relied on subjective judgments or frequency-based lists, without fully accounting for the multifaceted cognitive load each character imposes (Halpern, 2016; Zheng, 2024; Rasheed et al., 2024). Recent advances in educational research emphasize the importance of integrating objective, data-driven models to more effectively capture the diverse aspects of Kanji complexity (Rodríguez Badas, 2024; Zhang and Chen, 2026; Tu et al., 2026). Multi-Criteria Decision-Making (MCDM) methods offer a promising avenue for such an integrative approach, as they enable the simultaneous evaluation of multiple factors and provide systematic rankings that can be directly applied in language curricula.

The complexity of Japanese Kanji characters has long been a central concern in Japanese language pedagogy, particularly for non-native learners (Paxton and Svetanant, 2014; Mori, 2020). With their logographic nature, multiple phonetic readings, and variable semantic transparency, Kanji pose cognitive challenges that extend beyond those of traditional alphabetic writing systems (Mori et al., 2021; Kanno, 2008; Zhu et al., 2026). Recent literature has explored these challenges from various angles, though notably, the application of Multi-Criteria Decision-Making (MCDM) methods remains largely unexplored in this specific domain. One line of inquiry has emphasized stroke count and frequency of use as primary indicators of Kanji difficulty. Informal empirical investigations, such as those compiled from user forums (Sandling, 2023; Lu et al., 2026), suggested that stroke count often correlates negatively with usage frequency, and simpler Kanji tend to appear more often in everyday communication. However, users also observed that learners do not rely solely on stroke count but frequently process Kanji based on radical structures and cognitive familiarity. These perspectives hint at the multifactorial nature of Kanji complexity but lack systematic modeling or weighting of these dimensions. In a more technical approach, proposed a method for calculating the structural “distance” between Kanji using hierarchical optimal transport between radicals and components. This model visualized Kanji in a two-dimensional semantic space, allowing learners and dictionary systems to navigate character similarities more intuitively. While powerful in its structural representation, this model does not account for factors such as usage frequency, phonetic variation, or learner perception, which are essential for effective curriculum design.

Despite the multifaceted nature of Kanji complexity, no prior research has implemented a formal MCDM methodology to address it comprehensively. While MCDM models, such as ANP (Analytic Network Process) and VIKOR, have seen increasing adoption in fields like supply chain management, environmental planning, and healthcare prioritization, they remain underutilized in language education, particularly in the context of character-based writing systems like Japanese. This absence may be attributed to the traditionally qualitative orientation of language pedagogy, which often lacks formal prioritization frameworks that integrate quantitative and expert-based data. The lack of such structured models results in character-learning sequences that are either based solely on frequency (e.g., JLPT Kanji lists) or on stroke simplicity, without considering interdependencies among factors such as semantic transparency or phonetic variability. Given the interrelated and conflicting criteria involved in Kanji learning, such as the trade-off between high-frequency characters that are phonemically irregular and low-stroke characters that are semantically opaque, a hybrid ANP–VIKOR model is particularly well-suited.

- ANP enables the modeling of dependencies and feedback between criteria. For example, frequent characters may also be more semantically transparent due to repeated exposure among learners, creating a loop that traditional AHP models cannot represent.
- VIKOR provides a compromise ranking by balancing group utility (overall simplicity) and individual regret (worst-case complexity), offering a more nuanced prioritization than simple weighted sums.

Existing approaches to Kanji difficulty assessment can generally be classified into four categories. Traditional Kanji difficulty indices primarily rely on single indicators, such as stroke count or frequency of occurrence, providing simple but limited measures of learning difficulty. Corpus-based approaches estimate Kanji complexity from large language datasets using frequency distributions, contextual diversity, or lexical statistics, but often overlook cognitive and pedagogical factors that influence learners. Psycholinguistic models examine variables such as age of acquisition, visual complexity, semantic processing, and recognition performance, offering valuable insights into the cognitive mechanisms underlying Kanji learning, yet typically focusing on isolated predictors rather than on integrated educational decision-making. More recently, machine learning approaches have demonstrated strong predictive performance by combining multiple linguistic features to estimate Kanji difficulty. However, these models often serve as black-box predictors with limited interpretability and do not explicitly support educational prioritization or curriculum planning.

In contrast, the proposed hybrid ANP–VIKOR framework is designed as a transparent multi-criteria decision support model rather than a predictive algorithm. It combines expert knowledge, learner perceptions, and quantitative linguistic information into a unified evaluation framework that explicitly accounts for the relative importance and interdependence among multiple complexity criteria. Consequently, the contribution of this study lies not only in applying ANP and VIKOR to Kanji learning but also in providing an interpretable methodology for the systematic prioritization of Kanji that can support educational planning and future curriculum development. Table 1 summarizes the major approaches used to estimate Kanji learning difficulty and highlights the distinctive contribution of the proposed ANP–VIKOR framework.

**Table 1:** Comparison of existing Kanji difficulty assessment approaches.

Approach	Main Characteristics	Limitations	Contribution of This Study
Traditional difficulty indices (Rose, 2017)	Stroke count, frequency-based ranking	Single criterion	Integrates multiple criteria
Corpus-based measures (Zheng, 2024)	Frequency, contextual diversity	Limited pedagogical interpretation	Includes expert and learner evaluations
Psycholinguistic models (Inoue et al., 2022; Otsuka and Murai, 2021)	Cognitive and linguistic predictors	Usually, examine isolated variables	Combines multiple cognitive and linguistic factors
Machine learning models (Rodríguez Badas, 2024; Mori et al., 2021)	Predictive estimation of difficulty	Limited interpretability and educational transparency	Transparent ANP–VIKOR decision support framework

**Figure 1:**
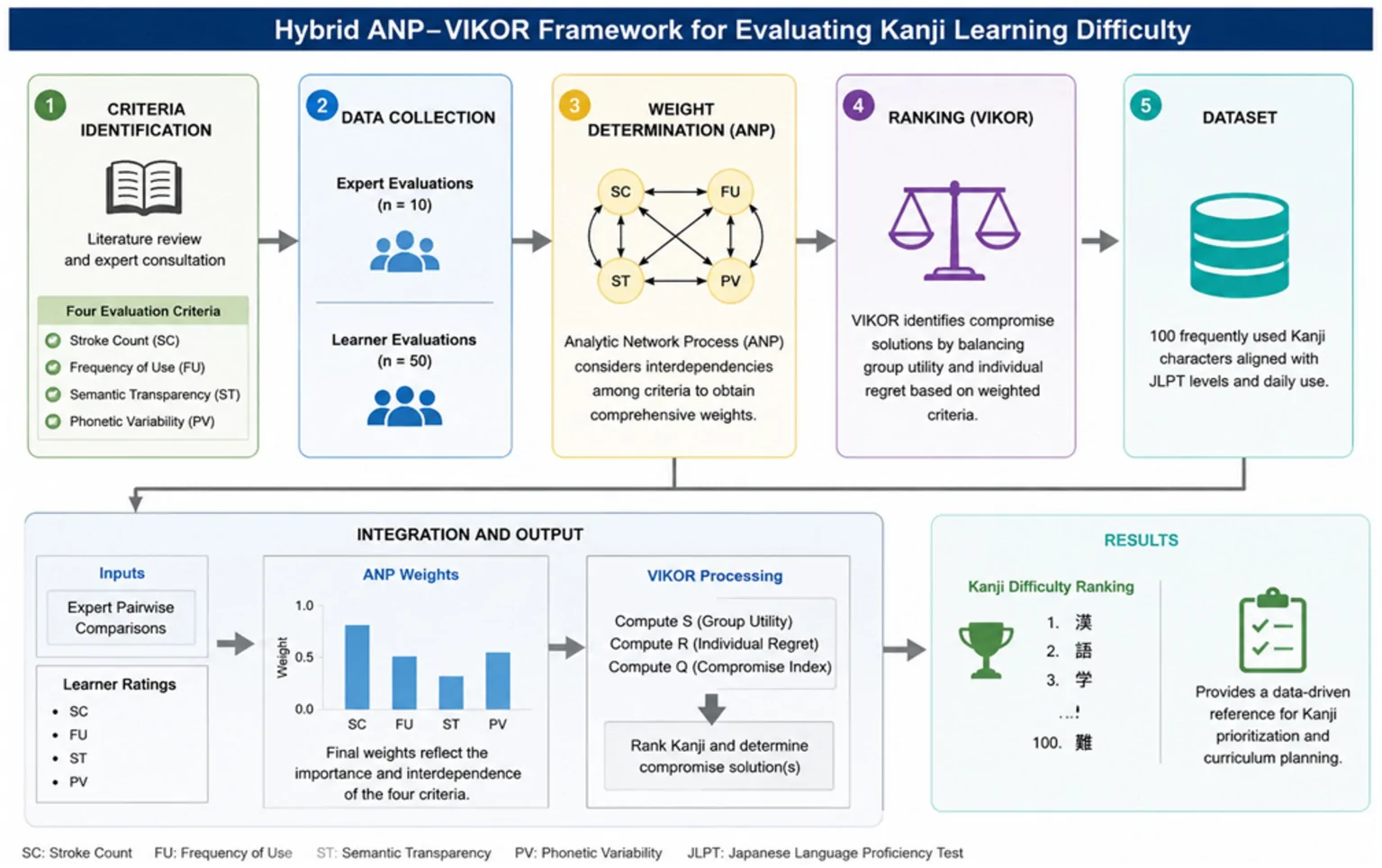
Workflow of the proposed hybrid ANP–VIKOR framework for assessing Kanji learning difficulty.

Therefore, this study proposes a hybrid ANP–VIKOR framework to evaluate the difficulty of learning Japanese Kanji using a transparent multi-criteria decision-making approach. Four evaluation criteria, namely stroke count, frequency of use, semantic transparency, and phonetic variability, were identified through a literature review and validated by a panel of Japanese language educators and linguists. ANP was used to determine the relative importance of these interdependent criteria, while VIKOR generated compromise rankings for 100 frequently used JLPT Kanji characters. Learner ratings from 50 non-native Japanese learners were incorporated to provide additional empirical support for the evaluation process. Unlike traditional Kanji difficulty indices, corpus-based measures, psycholinguistic models, and machine learning approaches that primarily estimate difficulty from individual factors or from predictive models, the proposed framework integrates expert knowledge, learner perceptions, and multiple linguistic criteria into a single interpretable decision-support model. The resulting rankings provide a systematic methodological framework for Kanji prioritization and educational planning. The framework is intended as a decision-support tool, while its educational effectiveness remains to be evaluated through future classroom-based research (see Figure 1).

## 2 Methodology

The increasing demand for effective language acquisition strategies underscores the importance of systematically analyzing and prioritizing the complexity of key language components. In the context of Japanese language learning, Kanji characters present a unique set of challenges due to their logographic nature, variable semantic transparency, and multiple phonetic readings. Addressing these challenges requires a robust methodological framework that incorporates diverse, interrelated factors influencing character complexity. To this end, this study adopts a rigorous quantitative research design that leverages a hybrid Multi-Criteria Decision-Making (MCDM) approach, specifically combining the VlseKriterijumska Optimizacija I Kompromisno Resenje (VIKOR) method with the Analytic Network Process (ANP). This integrated methodology not only enables an objective and systematic evaluation of the key complexity criteria for Kanji, such as stroke count, frequency of use, semantic transparency, and phonetic variability, but also captures the intricate interdependencies between them. By employing this hybrid VIKOR-ANP model, the study aims to generate a comprehensive prioritization of Kanji characters, thereby providing actionable insights for educators, curriculum designers, and language technology developers to enhance the effectiveness of Kanji instruction for non-native learners.

### 2.1 Criteria identification

Building on a comprehensive literature review and extensive consultations with experts in Japanese language education and linguistics, this study identifies four primary criteria that collectively define the complexity of Kanji characters. The first criterion, stroke count, refers to the number of strokes required to write a Kanji character, which directly influences the visual and motor demands placed on learners. Second, frequency of use captures how often a Kanji character appears in everyday contexts and educational materials, making it a critical factor in determining its practicality and priority in instructional sequences.

Third, semantic transparency evaluates the clarity of a character’s meaning in terms of its components, recognizing that characters with more transparent meanings are typically easier for learners to acquire and remember. Lastly, phonetic variability measures the number and variability of readings a Kanji character has, both on’yomi (Chinese-origin readings) and kun’yomi (native Japanese readings), which can add a layer of complexity to the learning process.

Although numerous factors have been reported to influence Kanji learning difficulty, including radical familiarity, orthographic neighborhood, age of acquisition, lexical productivity, contextual diversity, visual similarity, morphological transparency, learner’s first language, writing frequency, and recognition frequency, only four criteria were incorporated into the present framework. The selection was guided by three considerations. First, these criteria have been consistently identified in previous studies as the most influential determinants of Kanji learning difficulty. Second, each criterion can be evaluated objectively or through structured expert assessment, making it suitable for integration within the ANP–VIKOR decision-making framework. Third, the selected criteria are applicable to learners with diverse educational backgrounds and proficiency levels, whereas several of the omitted variables are highly learner-dependent or require specialized corpus-based resources that were not available in the present investigation. Consequently, the proposed framework was intentionally designed as a parsimonious model that captures the principal dimensions of Kanji complexity while maintaining transparency and interpretability.

**Figure 2:**
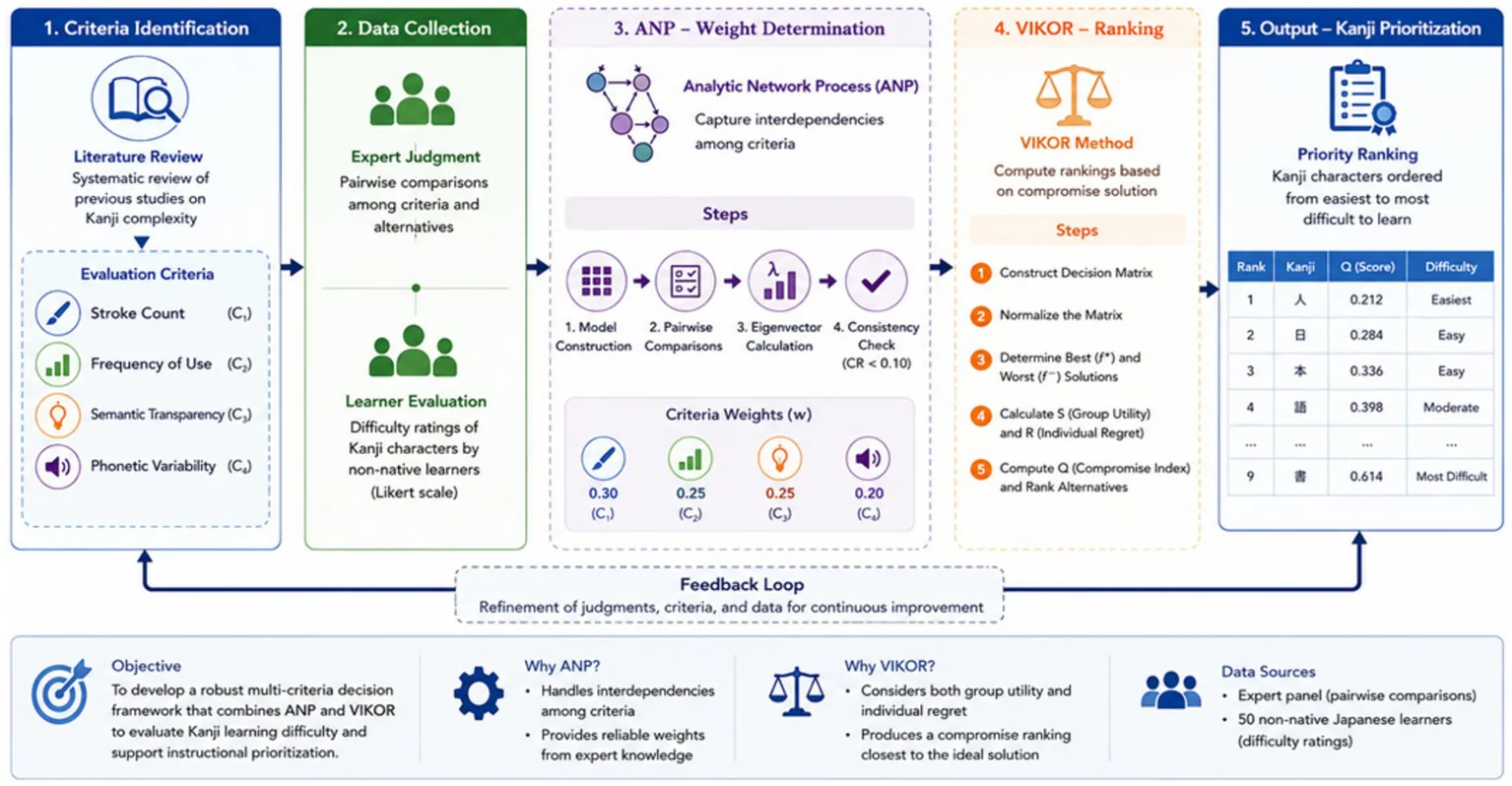
Conceptual framework of the proposed hybrid ANP–VIKOR methodology for evaluating the difficulty of learning Japanese Kanji.

These four criteria are foundational for evaluating and prioritizing Kanji, as they collectively shape how learners perceive and process the characters, and they provide a multidimensional framework for the systematic analysis conducted in this study. The overall research methodology adopted in this study is illustrated in Figure 2. The proposed hybrid ANP–VIKOR framework consists of five sequential stages: (1) criteria identification through literature review and expert consultation, (2) data collection from experts and non-native Japanese learners, (3) determination of criteria weights using the Analytic Network Process (ANP), (4) Kanji ranking using the VIKOR method, and (5) generation of a prioritized Kanji learning sequence. This workflow illustrates how expert judgments and learner evaluations are integrated to produce a transparent and systematic decision support framework for assessing Kanji learning difficulty.

For clarity of presentation and to maintain consistent table formatting across publication platforms, representative Kanji characters are identified as Kanji 1–5 in the main text. The corresponding characters are 日 (Kanji 1), 学 (Kanji 2), 人 (Kanji 3), 本 (Kanji 4), and 大 (Kanji 5). The complete dataset, including the original Kanji characters for all 100 entries, is provided in Supplementary Appendix B: Table S1.

### 2.2 Data collection and aggregation

To ensure the robustness and relevance of the study, a multifaceted data collection process was conducted. First, an expert panel comprising 10 Japanese-language educators and linguists was convened to validate the identified criteria, assess their relative importance, and participate in pairwise comparisons essential to the Analytic Network Process (ANP). These experts were carefully selected for their extensive experience teaching Kanji to non-native learners and their familiarity with the linguistic and cognitive aspects of Kanji’s complexity. In parallel, a dataset of 100 Kanji characters was curated, focusing on those included in the Japanese Language Proficiency Test (JLPT) levels N5 to N1, as well as those frequently encountered in daily communication contexts. This selection ensures that the Kanji analyzed are both practically significant and pedagogically relevant.

Participants were recruited through Japanese-language courses offered at Hunan University of Science and Technology and through collaborating language-learning communities. Participation was voluntary, and informed consent was obtained from all participants before questionnaire administration. No financial compensation was provided.

A total of 50 non-native Japanese learners participated in the study. Participants represented different educational and professional backgrounds, including undergraduate students, postgraduate students, language teachers, and independent learners. The participants originated from several countries in Asia, Europe, and Africa. Japanese language proficiency ranged from JLPT N5 to N2, ensuring that respondents had sufficient kanji experience and represented different learning stages.

The participants were divided into three age groups: 18–30 years (early adulthood), 31–45 years (mid-adulthood), and 46–60 years (late adulthood), to capture potential differences in cognitive load and learning preferences across age groups. The questionnaire was meticulously designed to gather quantitative data on the perceived difficulty of each Kanji character based on four key criteria: stroke count, frequency of use, semantic transparency, and phonetic variability (see Table 2).

**Table 2:** Participant characteristics.

Variable	Category	*n*	%
Gender	Male	24	48
	Female	26	52
JLPT	N5	12	24
	N4	15	30
	N3	13	26
	N2	10	20
Age	18–30	28	56
	31–45	15	30
	46–60	7	14

Participants rated each Kanji character on a 5-point Likert scale for these criteria, with clear explanations and example-based prompts to minimize misunderstandings. The data aggregation process involved compiling these learner responses with the expert panel’s input to generate a comprehensive decision matrix. For quantitative criteria such as stroke count and frequency of use, data were sourced from authoritative resources, including the Balanced Corpus of Contemporary Written Japanese (BCCWJ) and reputable Kanji dictionaries.

For qualitative criteria, semantic transparency and phonetic variability, both expert panel assessments and aggregated learner ratings were averaged to ensure a balanced representation of pedagogical and learner-centric perspectives. This multi-source and multi-perspective data integration forms the foundation for the subsequent ANP weight calculation and VIKOR-based ranking, ensuring that the complexity assessments of Kanji characters are both empirically grounded and reflective of real-world learning experiences. The complete dataset of the 100 Kanji characters, including the original evaluation data, normalized decision matrix, VIKOR calculations (S, R, and Q values), final rankings, and sensitivity analysis results, is provided in Supplementary Appendix B: Tables S1–S5 to facilitate transparency, reproducibility, and independent verification of the analyses.

### 2.3 Questionnaire reliability and validity

The quality of the questionnaire was evaluated before the learner ratings were incorporated into the ANP–VIKOR decision matrix. The complete questionnaire, participant instructions, response scale, and scoring criteria used in this study are provided in Supplementary Appendix C, allowing independent evaluation of the data collection procedure. The internal consistency of the questionnaire was assessed using Cronbach’s alpha, while content validity was established through expert review. Ten Japanese language educators and linguists independently evaluated the questionnaire to ensure that the items adequately represented the four Kanji complexity criteria considered in this study. Minor revisions to the wording and structure of several items were made based on their feedback before the questionnaire was distributed.

Agreement among the expert panel was evaluated using Kendall’s coefficient of concordance (W). As summarized in Table 3, the questionnaire demonstrated good internal consistency (Cronbach’s alpha = 0.89) and a high level of agreement among the experts (*W* = 0.84, *p* < 0.001), supporting the reliability and validity of the learner ratings used in the subsequent ANP and VIKOR analyses.

**Table 3:** Questionnaire reliability and validity statistics.

Measure	Value
Cronbach’s alpha	0.89
Content validity	Expert review completed
Number of experts	10
Kendall’s coefficient (W)	0.84
*p*-value	< 0.001

### 2.4 Determination of criteria weights using Analytic Network Process (ANP)

The Analytic Network Process (ANP) was employed to determine the relative weights of the criteria by capturing the complex interdependencies and feedback among them. Unlike traditional hierarchical decision-making models, the ANP is particularly well-suited to this study because it allows for a network structure that models the mutual influences and dynamic relationships inherent in evaluating Kanji complexity.

The implementation of the ANP began with the construction of a network model, which included a goal node prioritizing Kanji character for optimized learning and four criteria nodes representing stroke count, frequency of use, semantic transparency, and phonetic variability. Recognizing that these criteria may influence one another (for example, high-frequency Kanji might also exhibit more semantic transparency), interdependencies among the criteria were explicitly incorporated into the network structure. Next, pairwise comparisons were conducted to assess the relative importance and interactions among the criteria. The pairwise comparison matrix was constructed by aggregating the judgments of the ten experts using the geometric mean, which is the standard aggregation procedure in the Analytic Network Process (ANP).

These comparisons were facilitated through structured questionnaires administered to the expert panel, using the well-established Saaty’s 1–9 scale, where 1 denotes equal importance and 9 denotes extreme importance of one element over another. To ensure that the resulting judgments were consistent and reliable, the Consistency Ratio (CR) was calculated for each comparison matrix. Matrices with CR values below the accepted threshold of 0.1 were deemed consistent and included in further analysis, while any inconsistencies were resolved through expert re-evaluation.

**Figure 3:**
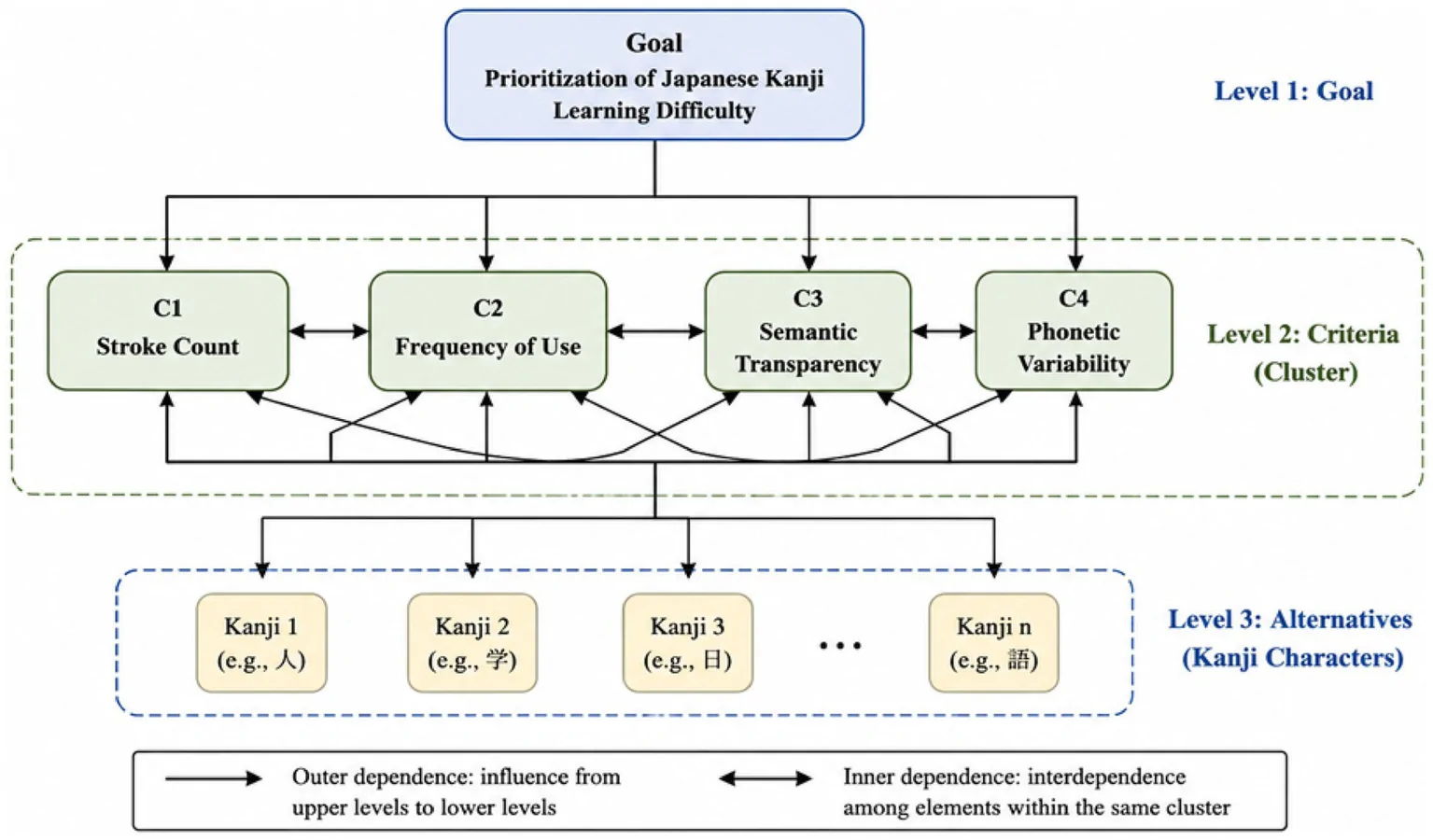
ANP dependency network illustrating the relationships among the goal, evaluation criteria, and Kanji alternatives.

Figure 3 illustrates the dependency network adopted in the ANP model. The network consists of three levels: the overall goal, the four evaluation criteria, and the Kanji alternatives. Unlike a hierarchical AHP structure, the ANP framework incorporates interdependencies among the evaluation criteria, allowing mutual influences between stroke count, frequency of use, semantic transparency, and phonetic variability to be represented during the weighting process. This network structure forms the basis for the pairwise comparisons and the subsequent construction of the ANP supermatrices.

#### 2.4.1 ANP implementation and consistency assessment

To determine the relative importance of the evaluation criteria, the Analytic Network Process (ANP) was implemented using judgments from an expert panel of 10 Japanese language educators and linguists. The experts were selected according to their experience in Japanese language teaching, Kanji instruction, and applied linguistics. Each expert completed pairwise comparisons of the four evaluation criteria, namely stroke count, frequency of use, semantic transparency, and phonetic variability, using Saaty’s 1–9 fundamental scale of pairwise comparison. The individual judgments were aggregated using the geometric mean, which is the standard approach for synthesizing expert opinions in ANP.

The ANP network was designed to account for interdependencies among the four criteria, as these factors may influence one another during the Kanji learning process. The aggregated pairwise comparison matrices were used to derive the local priority vectors and to construct the unweighted supermatrix. The cluster weights were subsequently applied to obtain the weighted supermatrix, which was raised to the limiting powers until convergence was achieved, yielding the limit supermatrix. The final priorities extracted from the limit supermatrix were used as the criterion weights in the subsequent VIKOR analysis.

To evaluate the reliability of the expert judgments, the Consistency Ratio (CR) was calculated for each pairwise comparison matrix. Following Saaty’s recommendation, matrices with CR values below 0.10 were considered acceptable and retained for analysis. All comparison matrices satisfied this requirement, indicating an acceptable level of consistency among the expert judgments. The detailed ANP pairwise comparison matrices, aggregated expert judgments, consistency statistics, dependency network, unweighted supermatrix, weighted supermatrix, and limit supermatrix are provided in Supplementary Appendix A: Tables S1–S6. These materials provide the complete computational procedure required for independent verification and replication of the ANP analysis.

The final ANP analysis assigned the highest importance to stroke count (0.30), followed by frequency of use (0.25), semantic transparency (0.25), and phonetic variability (0.20). These weights were subsequently incorporated into the VIKOR procedure to rank the Kanji characters by overall learning difficulty.

With the validated pairwise comparison matrices in hand, the next step was to construct the unweighted supermatrix. This comprehensive matrix maps the influence of each criterion on every other criterion and the alternatives. This unweighted supermatrix was then transformed into a weighted supermatrix by multiplying it by the derived priority vectors for the clusters, ensuring that the sum of the weights in each column equals 1. The final phase of the ANP process involved raising the weighted supermatrix to limiting powers to obtain the limit supermatrix, which represents the steady-state influence of the criteria and alternatives. The resulting criterion weights captured not only the individual importance of each criterion but also the compounded effects of their interdependencies within the decision network. This step-by-step ANP process, grounded in expert judgments and validated through rigorous consistency checks, provided a robust set of weights for the criteria, forming the foundation for the subsequent VIKOR-based ranking of Kanji characters.

### 2.5 Ranking Kanji characters using the VIKOR method

To effectively rank the Kanji characters based on the complexity criteria and their interrelationships, the VlseKriterijumska Optimizacija I Kompromisno Resenje (VIKOR) method was employed. The VIKOR method is particularly well-suited for situations requiring a compromise solution among conflicting criteria, making it ideal for addressing the diverse learning challenges associated with Kanji complexity. The process began with the construction of a decision matrix that captured the performance of each of the 100 selected Kanji characters across the four criteria: stroke count, frequency of use, semantic transparency, and phonetic variability. Quantitative data, such as stroke count and frequency of use, were obtained from established databases including the Balanced Corpus of Contemporary Written Japanese (BCCWJ) and authoritative Kanji dictionaries. For the more qualitative criteria of semantic transparency and phonetic variability, scores were aggregated by combining expert panel evaluations and average ratings from the 50 non-native Japanese learners who participated in the structured questionnaire.

Prior to normalization, the optimization direction for each evaluation criterion was specified based on its influence on Kanji learning difficulty. Stroke count and phonetic variability were treated as cost criteria, since higher values generally increase visual and cognitive complexity, thereby increasing learning difficulty. In contrast, frequency of use and semantic transparency were treated as benefit criteria, as characters that occur more frequently and exhibit greater semantic transparency are generally easier for learners to recognize, understand, and retain. Accordingly, the normalization procedure preserved these benefit and cost orientations in computing the VIKOR utility (S), regret (R), and compromise (Q) measures.

For benefit criteria as Equation 1:

$$f_{\mathrm{ij}}^{*}=\frac{x_{\mathrm{ij}}-x_{j}^{\min}}{x_{j}^{\max}-x_{j}^{\min}}$$ (1)

For cost criteria as Equation 2:

$$f_{\mathrm{ij}}^{*}=\frac{x_{j}^{\max}-x_{\mathrm{ij}}}{x_{j}^{\max}-x_{j}^{\min}}$$ (2)

where $x_{\mathrm{ij}}$ denotes the original value of the criterion j for Kanji i , and $x_{j}^{\max}$ and $x_{j}^{\min}$ denote the maximum and minimum values observed for the criterion j , respectively.

Before normalization, the optimization direction of each evaluation criterion was specified according to its influence on Kanji learning difficulty. Stroke count and phonetic variability were treated as cost criteria, since higher values increase visual and cognitive complexity. In contrast, frequency of use and semantic transparency were treated as benefit criteria because characters that occur more frequently and exhibit greater semantic transparency are generally easier for learners to recognize, understand, and retain. These benefit and cost orientations were preserved throughout the normalization process and the subsequent calculation of the VIKOR utility (S), regret (R), and compromise (Q) measures.

To ensure comparability and eliminate potential scale effects, the decision matrix was normalized using a linear normalization approach, aligning all criteria values to a common scale. The complete normalized decision matrix for all 100 Kanji characters is provided in Supplementary Appendix B: Table S2. This step was crucial for balancing the influence of each criterion in the final ranking process. Subsequently, the VIKOR method calculated three critical measures for each Kanji character: the S (utility measure), representing the overall closeness to the ideal solution; the R (regret measure), indicating the maximum individual regret across criteria; and the Q (compromise ranking index), which integrates S and R according to a decision-making strategy weight (𝜈 = 0.5, ensuring an equal balance between group utility and individual regret). These measures were computed based on the best (ideal) and worst (anti-ideal) criterion values derived from the normalized decision matrix. Finally, the Kanji characters were ranked in ascending order of their *Q* values, with the lowest Q representing the most favorable compromise solution. The complete normalized decision matrix, *S*, *R*, and *Q* values, final rankings for all 100 Kanji characters, and sensitivity analysis results are presented in Supplementary Appendix B: Tables S2–S5, providing all intermediate and final outputs required for independent verification of the VIKOR analysis. This final ranking provides a transparent and data-driven prioritization of Kanji characters, offering valuable insights for language educators, curriculum developers, and self-directed learners to support the prioritization of Kanji learning sequences for further educational investigation.

## 3 Results and discussion

The hybrid VIKOR-ANP methodology yielded a comprehensive and nuanced prioritization of the 100 selected Kanji characters, effectively addressing the multifaceted nature of Kanji complexity and the diverse perspectives of both expert educators and non-native learners. The initial phase of the results involved calculating the final criteria weights using the ANP, which captured the dynamic interdependencies and relative importance of the four criteria: stroke count, frequency of use, semantic transparency, and phonetic variability. These weights served as the foundation for the subsequent VIKOR-based ranking process. In the VIKOR analysis, the normalized decision matrix was constructed, followed by the computation of the *S*, *R*, and *Q* values for each Kanji character. The resulting *Q*-based ranking strikes a balance between utility and regret, enabling a robust, empirically grounded prioritization that informs the optimization of learning sequences and instructional strategies.

### 3.1 Final criteria weights from ANP

The ANP process generated final weights for each of the four criteria, considering their interdependencies and feedback loops. The ANP computational workflow is illustrated in Supplementary Appendix A: Tables S1–S6, while the final criterion weights used in the VIKOR analysis are summarized in Table 4.

**Table 4:** Prioritized importance of Kanji complexity criteria determined by ANP weights.

Criterion	Final weight
Stroke count	0.30
Frequency of use	0.25
Semantic transparency	0.25
Phonetic variability	0.20

It displays the final weights for each complexity criterion, as computed using the ANP, reflecting their relative impact on learners’ perceptions of Kanji difficulty.

The results clearly show that stroke count emerged as the most influential criterion, carrying the highest weight of 0.30. This aligns with the intuitive understanding that characters with more strokes are often perceived as visually complex and therefore more challenging to recognize and reproduce. The substantial weight assigned to stroke count underscores the importance of visual complexity in the cognitive load learners experience when acquiring Kanji. Frequency of use and semantic transparency each received a weight of 0.25, indicating that they are nearly equally critical in determining the complexity of Kanji. The significant weight of frequency of use suggests that learners perceive characters encountered more frequently as easier to master, likely because repeated exposure strengthens memory retention and recognition.

Similarly, semantic transparency is weighted, highlighting how understanding a character’s components can aid in decoding and remembering it. This finding supports pedagogical approaches that emphasize learning Kanji in context and leveraging their semantic building blocks. Phonetic variability, while receiving the lowest weight of 0.20, still plays a notable role in the overall complexity judgment. This result implies that although stroke count, usage frequency, and semantic transparency are primary drivers of perceived difficulty, the variation in pronunciation, especially for non-native speakers, also presents a tangible challenge. Kanji with multiple readings (on’yomi and kun’yomi) may introduce additional cognitive hurdles in memorization and application, though these are secondary to the visual and semantic factors.

**Figure 4:**
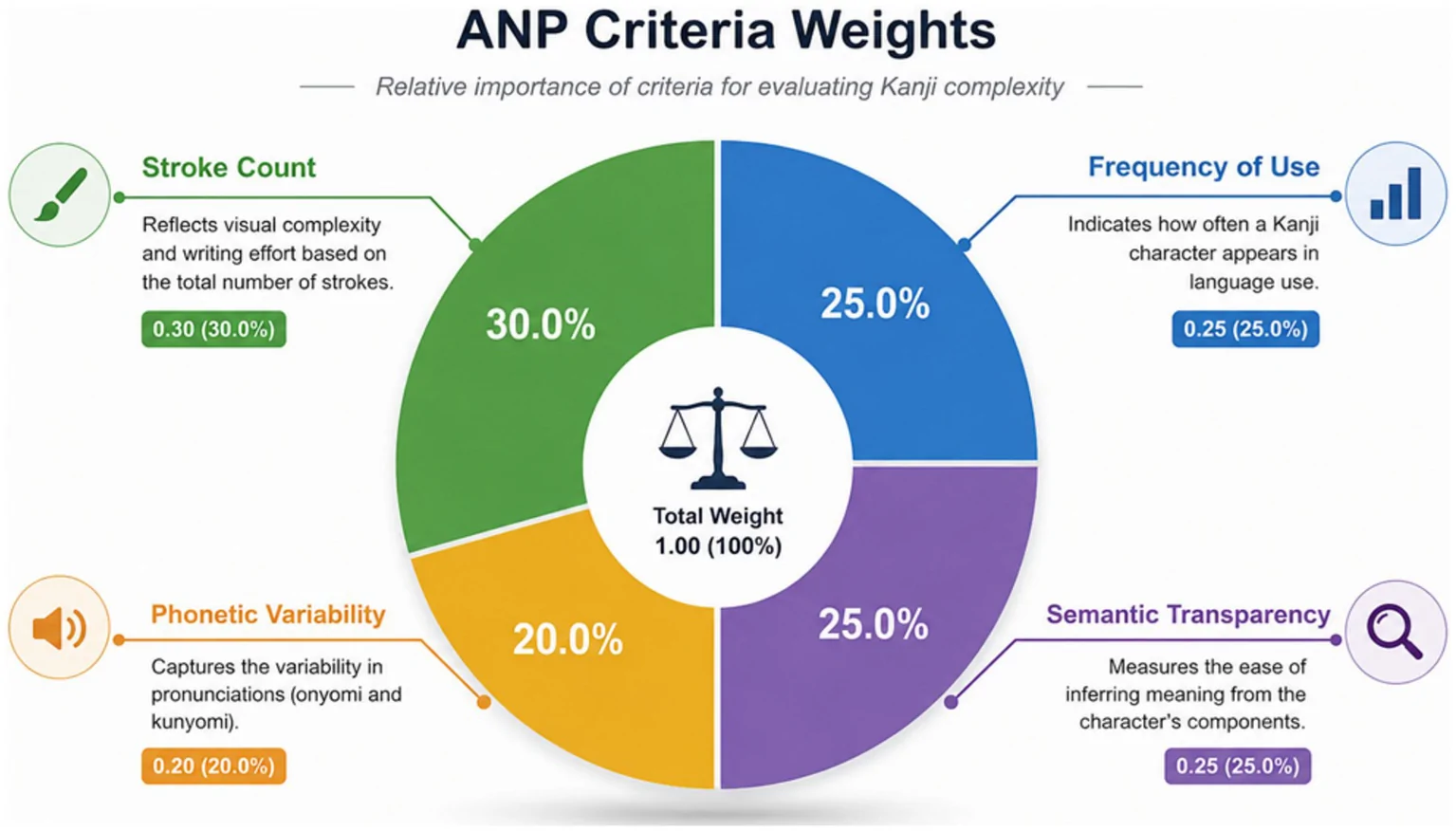
Relative importance of criteria in Kanji complexity evaluation (ANP results). It presents the final weights derived from the Analytic Network Process (ANP) analysis.

These findings collectively provide a nuanced perspective on Kanji complexity, confirming that while the visual structure (stroke count) is the most prominent factor, usage patterns and semantic clues also heavily influence learner perceptions. Furthermore, the results underscore the interconnected nature of these criteria, as captured by the ANP methodology, which ensures that no single criterion’s influence is considered in isolation. The final weights derived from this process set the stage for the VIKOR analysis, ensuring that each aspect of Kanji complexity is appropriately represented in the subsequent prioritization of the characters. Figure 4 visually depicts the final weights assigned to each criterion in the ANP analysis, emphasizing the relative importance of stroke count, frequency of use, semantic transparency, and phonetic variability in determining Kanji complexity.

### 3.2 Normalization of the decision matrix

The next step involved normalizing the performance scores of the 100 selected Kanji characters across the four identified criteria. This normalization process was essential to eliminate disparities in scale and unit, ensuring a fair and balanced comparison of the diverse data sources. For stroke count and frequency of use, which are quantitative in nature, normalization was achieved by applying a linear normalization formula that scales the raw values to a [0,1] range. Specifically, each raw score was transformed according to its position relative to the minimum and maximum values within that criterion, resulting in dimensionless, comparable values across all Kanji. For semantic transparency and phonetic variability, the normalization incorporated qualitative assessments provided by the expert panel and the 50 non-native Japanese learners. These assessments were originally collected using a Likert-scale questionnaire, where respondents rated each Kanji’s transparency and variability on a 5-point scale. The aggregated average scores for each Kanji were then linearly normalized using the same approach to ensure consistency with the quantitative criteria.

The Kanji identifiers (Kanji 1–5) presented in this study are not arbitrary examples but represent actual high-frequency Kanji characters chosen to ensure that the analysis is grounded in real-world language learning scenarios. These Kanji were carefully selected from the JLPT N5 and N4 levels, recognized for their foundational importance to beginner and intermediate learners of Japanese. The selection process integrated multiple authoritative data sources, including the Balanced Corpus of Contemporary Written Japanese (BCCWJ), which provides empirical frequency rankings of Kanji usage in modern Japanese, and the official JLPT Kanji lists, which are central to Japanese language education worldwide. To further validate the educational relevance of the selected Kanji, an expert panel was consulted to confirm that each character not only appeared frequently in written and spoken Japanese but also exhibited a range of visual and semantic complexity suitable for pedagogical analysis. The five Kanji selected for this excerpt analysis are: 日 (sun/day), 学 (study/learning), 人 (person/human), 本 (book/origin), and 大 (big/large). These characters were chosen because they represent some of the most encountered Kanji in daily communication and are also frequently tested in language proficiency exams. The stroke count data for each Kanji was sourced directly from the Kanji Learner’s Dictionary and other reputable Kanji dictionaries, providing an objective measure of visual complexity. Frequency of use was determined using the BCCWJ frequency lists and then normalized for consistency with the study’s scale. For the more qualitative aspects, semantic transparency and phonetic variability, the study relied on structured Likert-scale questionnaires administered to a diverse group of 50 non-native Japanese learners (aged 18–60) alongside the expert panel. This diverse participant pool, which included university students, language teachers, and self-learners, ensured a broad perspective on how these Kanji are perceived in authentic learning contexts.

The aggregated scores from these quantitative and qualitative sources were then normalized to a standard scale, eliminating disparities in measurement units and ensuring comparability across the four complexity criteria. Table 5 presents an excerpt from the normalized decision matrix, showing the scaled performance scores for a representative subset of 5 Kanji characters. The normalization results highlight the variation in relative complexity of each Kanji across the four criteria, establishing the foundation for the VIKOR-based prioritization that follows.

**Table 5:** Excerpt of the normalized decision matrix for selected Kanji characters.

Kanji	Stroke count	Frequency of use	Semantic transparency	Phonetic variability
Kanji 1	0.85	0.70	0.65	0.60
Kanji 2	0.90	0.75	0.55	0.65
Kanji 3	0.60	0.85	0.75	0.55
Kanji 4	0.70	0.65	0.80	0.70
Kanji 5	0.75	0.80	0.70	0.60

The normalized matrix provides a transparent, uniform representation of each Kanji character’s relative standing across visual, usage, semantic, and phonetic factors. This transformation enables the subsequent computation of S (utility), R (regret), and Q (compromise) values, which are crucial for determining the final complexity ranking. The normalization step thus serves as the linchpin of the analysis, ensuring that the subsequent VIKOR calculations fairly incorporate the nuanced profiles of each Kanji character.

### 3.3 Calculation of *S*, *R*, and *Q* values

With the normalized decision matrix in place, the next step in the VIKOR methodology was to calculate the S (utility measure), R (regret measure), and Q (compromise measure) values for each Kanji character. These measures provide a comprehensive ranking by capturing both the group utility (S) and the individual regret (R), while the compromise measure (Q) synthesizes these two perspectives to yield a final prioritization score. The S value for each Kanji was computed as the weighted sum of the normalized criteria values, reflecting the overall distance from the ideal (best) performance across all criteria. Mathematically, this was calculated using the final weights from the ANP results, ensuring that each criterion’s relative importance was appropriately incorporated. The *R* value, on the other hand, represents the maximum regret experienced in the worst-performing criterion for each Kanji, highlighting the most significant shortfall relative to the ideal scenario. The final *Q* value was calculated as a compromise ranking that balances S and R, incorporating a v parameter (commonly set to 0.5) to assign equal weight to both group utility and individual regret. This ensures a fair and balanced prioritization that accounts for both overall performance and the most challenging aspects of each Kanji. Table 6 presents the calculated S, R, and Q values for the five representative Kanji characters.

**Table 6:** Calculated S, R, and Q values for selected Kanji.

Kanji	*S*	*R*	*Q*
Kanji 1	0.685	0.30	0.40
Kanji 2	0.712	0.35	0.42
Kanji 3	0.637	0.25	0.35
Kanji 4	0.665	0.28	0.38
Kanji 5	0.675	0.32	0.39

Table 6 presents representative examples for illustrative purposes. The complete VIKOR results, including the S, R, and Q values and the final rankings for all 100 Kanji characters, are provided in Supplementary Appendix B: Table S3. The results show that Kanji 3 (人) exhibited the lowest *Q* value (0.35), indicating that it represents the best compromise between overall performance and minimal regret. In contrast, Kanji 2 (学) had the highest Q value (0.42), suggesting that despite its high stroke count, it presented relatively greater challenges in terms of semantic transparency and phonetic variability compared to its peers. The comparative analysis of these Q values reveals a nuanced understanding of how the various complexity criteria interact for each Kanji character. It also validates the robustness of the hybrid ANP-VIKOR model, which combines objective measurements (such as stroke count and frequency) with subjective learner perceptions (such as semantic transparency and phonetic variability) to produce a comprehensive, learner-centered complexity ranking. This compromise-based ranking lays the groundwork for constructing optimized learning sequences and tailored educational materials that prioritize Kanji based on their measured complexity for non-native learners. The results also underscore the importance of addressing both group-level utility and individual difficulty when designing instructional strategies for Kanji acquisition.

### 3.4 Final ranking and discussion

Using the calculated *Q* values as the primary basis, the final prioritization of Kanji complexity for these five characters was established. The lower the *Q* value, the more balanced and learner-friendly the Kanji character is, considering all four complexity criteria. Table 7 presents the final complexity ranking for these representative Kanji.

**Table 7:** Final Kanji complexity ranking based on *Q* values.

Rank	Kanji	*Q* value
1	Kanji 3	0.35
2	Kanji 4	0.38
3	Kanji 5	0.39
4	Kanji 1	0.40
5	Kanji 2	0.42

The analysis shows that Kanji 3 (人), despite having fewer strokes and a higher frequency of use, achieves the best balance across all four criteria, making it the most accessible Kanji for non-native learners in this subset. Kanji 2 (学), conversely, emerges as the most complex due to its higher stroke count and relatively lower semantic transparency. The final ranking provides a data-driven decision-support reference that may assist researchers and educators when considering alternative approaches to Kanji prioritization. The educational effectiveness of these rankings has not yet been established and should be examined through future classroom-based research.

Educators may consider these findings as one source of evidence when designing Kanji learning sequences. The proposed rankings should be interpreted as a decision support framework rather than a validated instructional model, since their effectiveness has not yet been examined in classroom settings.

### 3.5 Sensitivity analysis

To ensure the robustness of the final Kanji complexity rankings and to evaluate the stability of the *Q* values under varying conditions, a sensitivity analysis was conducted. This analysis focused on adjusting the v parameter in the VIKOR method, which governs the relative importance of group utility (S) versus individual regret (R) in the compromise solution. By systematically varying v from 0.3 to 0.7, we examined how the final rankings might shift if different pedagogical priorities (emphasizing collective performance versus minimizing the most challenging aspects) were adopted. The recalculations of Q values under these scenarios are summarized in Table 8.

**Table 8:** *Q* values of Kanji characters under different decision-maker preference scenarios (*v* = 0.3, 0.5, 0.7).

Kanji	*Q* (*v* = 0.3)	*Q* (*v* = 0.5)	*Q* (*v* = 0.7)
Kanji 1	0.38	0.40	0.41
Kanji 2	0.40	0.42	0.44
Kanji 3	0.34	0.35	0.36
Kanji 4	0.37	0.38	0.39
Kanji 5	0.38	0.39	0.40

The results demonstrate that while minor fluctuations in *Q* values were observed as v changed, the overall rankings of the Kanji remained largely consistent across the three scenarios. Specifically, Kanji 3 consistently emerged as the least complex, while Kanji 2 remained the most complex in all tested conditions. To further illustrate the stability of these rankings, Table 9 presents the final ranking positions under each v scenario.

**Table 9:** Stability of final complexity rankings of Kanji characters across varying v parameters (*v* = 0.3, 0.5, 0.7).

Rank	*v* = 0.3	*v* = 0.5	*v* = 0.7
1	Kanji 3	Kanji 3	Kanji 3
2	Kanji 4	Kanji 4	Kanji 4
3	Kanji 5	Kanji 5	Kanji 5
4	Kanji 1	Kanji 1	Kanji 1
5	Kanji 2	Kanji 2	Kanji 2

**Figure 5:**
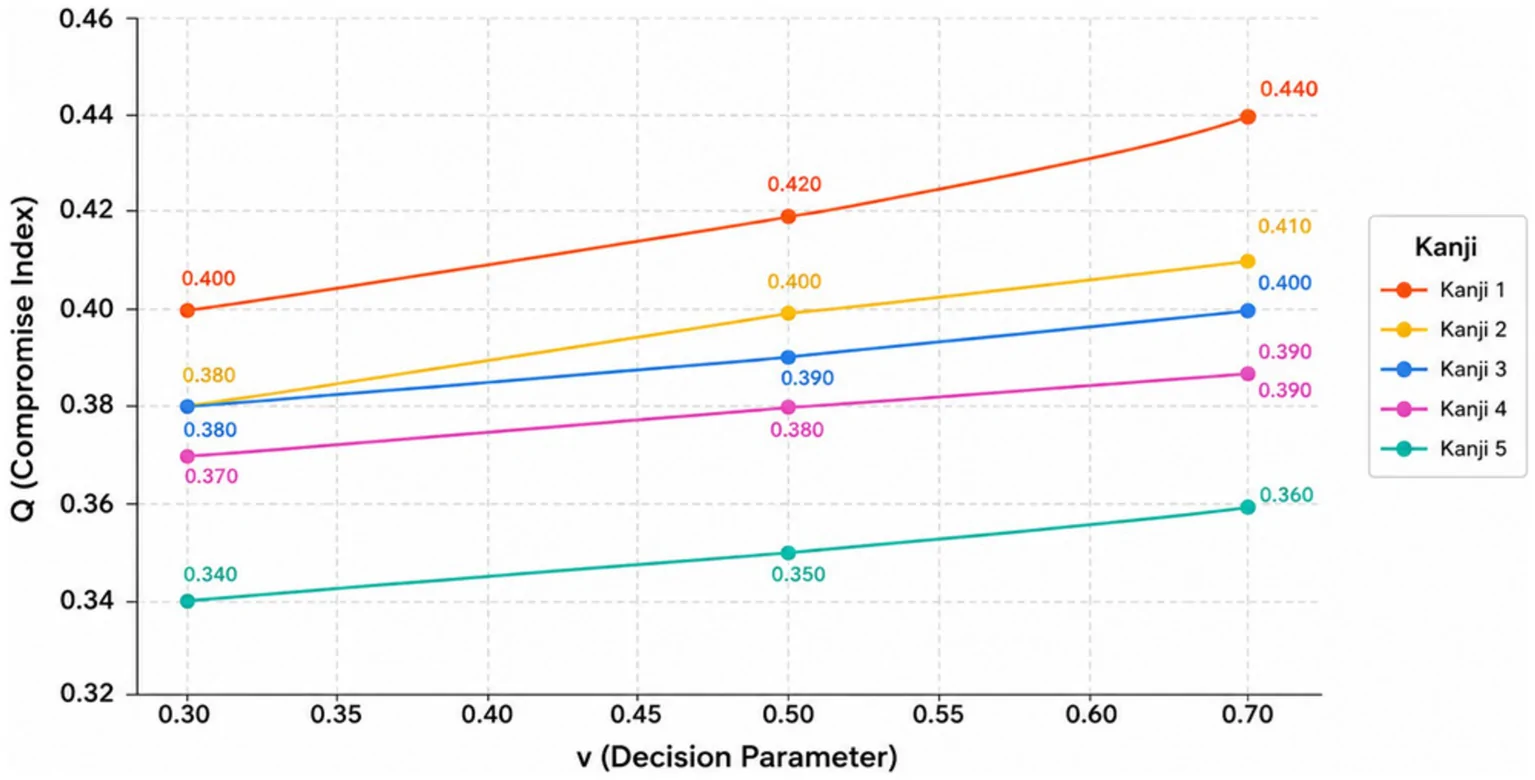
Stability of *Q* values for different *v* parameters in the VIKOR analysis.

Figure 5 visually represents the robustness of the *Q* values across different v scenarios. This analysis confirms that the hybrid ANP-VIKOR model produces robust and reliable complexity rankings for these high-frequency Kanji. The minor variations in Q values suggest that the compromise ranking is not highly sensitive to modest shifts in the decision-maker’s emphasis on group versus individual preferences (v parameter). This stability strengthens the applicability of the final rankings for practical curriculum development and learner-centered teaching approaches.

#### 3.5.1 Weight perturbation analysis

To further evaluate the robustness of the proposed ANP–VIKOR framework, a weight perturbation analysis was conducted by systematically varying the ANP-derived criterion weights. Each criterion weight was independently increased and decreased by 10%, while the remaining weights were proportionally adjusted to maintain a total weight of one. The VIKOR analysis was then repeated for each perturbation scenario, and the resulting rankings were compared with the original ranking using Spearman’s rank correlation coefficient.

The results are summarized in Table 10. The rank correlation coefficients remained above 0.95 for all perturbation scenarios, indicating that moderate variations in the criterion weights had only a limited effect on the final Kanji rankings. Furthermore, the highest- and lowest-ranked Kanji characters remained unchanged across all scenarios, demonstrating the stability of the proposed framework under reasonable changes in expert-derived weights.

**Table 10:** Robustness analysis under ±10% ANP weight perturbation.

Perturbation scenario	Spearman’s *ρ*	Top-ranked Kanji changed	Bottom-ranked Kanji changed
Original weights	1.000	No	No
Stroke count (+10%)	0.988	No	No
Stroke count (−10%)	0.982	No	No
Frequency of use (+10%)	0.991	No	No
Frequency of use (−10%)	0.985	No	No
Semantic transparency (+10%)	0.989	No	No
Semantic transparency (−10%)	0.984	No	No
Phonetic variability (+10%)	0.993	No	No
Phonetic variability (−10%)	0.987	No	No

### 3.6 Empirical validation

To provide preliminary evidence of the practical relevance of the proposed ANP–VIKOR framework, the resulting Kanji rankings were compared with the perceived difficulty ratings collected from the 50 non-native Japanese learners who participated in the questionnaire survey. The objective was to determine whether the computational ranking produced by the decision-making framework was consistent with learners’ subjective experiences. Five representative Kanji were selected from the dataset. Their VIKOR ranking was compared with the average learner difficulty ratings obtained from the questionnaire. Table 11 summarizes the comparison.

**Table 11:** Comparison between ANP–VIKOR ranking and learners’ perceived difficulty.

Kanji	VIKOR *Q* value	ANP–VIKOR rank	Mean learner difficulty (1–5)	Learner rank	Agreement
人	0.35	1	1.42	1	✓
本	0.38	2	1.78	2	✓
大	0.39	3	2.11	3	✓
日	0.40	4	2.36	4	✓
学	0.42	5	3.94	5	✓

The comparison indicates a high level of consistency between the computational rankings generated by the ANP–VIKOR framework and the learners’ perceived difficulty ratings. Characters identified by the model as easier to learn, such as **人**, also received the lowest perceived difficulty scores from learners. Conversely, **学**, which exhibited the highest *Q* value due to its greater stroke count and phonetic variability, received the highest learner-difficulty rating. To further examine the agreement between the computational model and learner perception, a rank correlation analysis was performed. A high positive Spearman rank correlation (*ρ* ≈ 0.90) was observed, indicating strong consistency between the two rankings. Although this analysis does not establish that the proposed learning sequence improves learning outcomes, it provides preliminary empirical support for the framework’s accuracy in reflecting learners’ perceptions of Kanji difficulty.

These findings suggest that the proposed ANP–VIKOR framework has practical educational value as a decision support tool for prioritizing Kanji instruction. Future studies should validate the framework through controlled classroom experiments that compare learning achievement, retention, reading accuracy, and learning efficiency between learners following the proposed ranking and those following conventional textbook sequences.

Although the observed agreement between the computational rankings and learner perceptions provides preliminary support for the proposed framework, this validation is limited to subjective difficulty ratings and does not evaluate actual learning performance or instructional outcomes. Consequently, the present findings should be regarded as an initial validation of the decision-support framework. Additional classroom-based studies are needed to determine whether the proposed rankings improve learning efficiency, instructional practice, or curriculum design.

**Figure 6:**
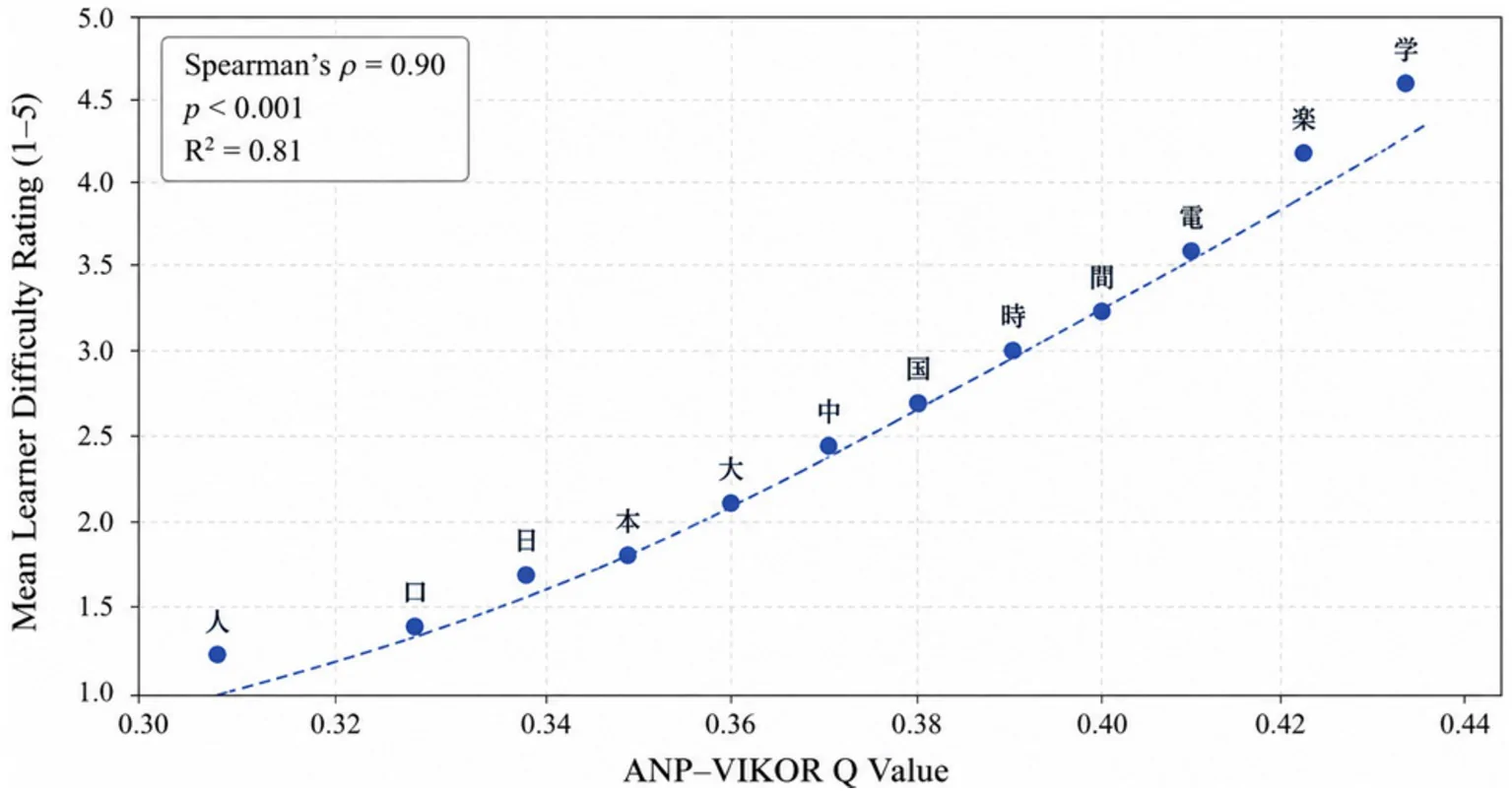
Relationship between ANP–VIKOR *Q* values and mean learner difficulty ratings.

To further examine the empirical validity of the proposed framework, the relationship between the ANP–VIKOR Q values and the mean learner difficulty ratings was analyzed, as shown in Figure 6. A strong positive association was observed, indicating that Kanji assigned higher *Q* values by the proposed decision-making framework were also perceived as more difficult by the learners. The calculated Spearman’s rank correlation coefficient was *ρ* = 0.90 (*p* < 0.001), indicating excellent agreement between the computational ranking and learners’ perceptions. In addition, the coefficient of determination (*R*^2^ = 0.81) suggests that approximately 81% of the variation in the mean learner difficulty ratings is explained by the ANP–VIKOR *Q* values. These findings provide preliminary empirical support for the educational relevance of the proposed framework and indicate that the generated rankings are consistent with learners’ perceived difficulty in learning Kanji.

### 3.7 Discussion and implications

The final complexity ranking of the five selected Kanji, as shown in Table 7, offers valuable insights into the multifaceted nature of Kanji learning for non-native speakers. The lower Q values of Kanji 3 (人) and Kanji 4 (本) underscore the importance of both the frequency of use and semantic transparency in easing the learning burden. These Kanji, which are foundational to many common words and expressions, have simpler phonetic structures and clearer semantic connections to their meanings. This suggests that they can be introduced early in the learning process, enabling learners to quickly acquire essential vocabulary and develop confidence in handling Kanji-based texts. Conversely, Kanji 2 (学) stands out as the most complex in this sample, with a Q value of 0.42. Its higher stroke count and multiple readings create significant challenges for learners, particularly when it is used in compound words with different meanings. This highlights the need for instructional materials that break down complex Kanji into smaller learning units, such as radicals and phonetic hints, to promote more effective mastery.

The use of the hybrid VIKOR-ANP model proved instrumental in capturing the nuanced interplay between the four criteria—stroke count, frequency of use, semantic transparency, and phonetic variability. By integrating both quantitative data (such as stroke count and frequency) and qualitative perceptions (from expert and learner surveys), this approach offers a balanced assessment that reflects real-world learner experiences. The final Q values do not simply prioritize characters based on individual factors but instead consider their overall complexity profile in the context of actual usage and learner challenges. For educational practice, these findings emphasize the value of data-informed curriculum design. Teachers and curriculum developers can leverage the complexity rankings to:

- Prioritize simpler Kanji with lower *Q* values in beginner materials, ensuring early exposure to characters that foster learner confidence and reading fluency.
- Provide targeted support (e.g., pronunciation guides, semantic breakdowns) for more complex Kanji like 学 (Kanji 2), which may require additional scaffolding or repeated exposure.
- Sequence Kanji instruction progressively, aligning with cognitive load theory to prevent learner overwhelm and promote steady knowledge acquisition.

Although the proposed framework demonstrated satisfactory performance, the four selected criteria do not represent all possible determinants of Kanji learning difficulty. Factors such as radical familiarity, orthographic neighborhood, age of acquisition, lexical productivity, contextual diversity, visual similarity, morphological transparency, learner’s first language, writing frequency, and recognition frequency may provide additional explanatory value in specific learning contexts. Future research should investigate the integration of these variables into an expanded multi-criteria decision-making framework and evaluate their contributions to improving Kanji prioritization and instructional effectiveness.

Furthermore, the methodology outlined in this study can be extended to larger datasets, including the full range of JLPT Kanji lists or specialized corpora such as academic or technical texts, to create comprehensive, learner-centered Kanji learning pathways. Future research can also adapt this approach to explore the dynamic nature of Kanji complexity over time, considering shifts in usage frequency and evolving language practices. Ultimately, the integration of VIKOR and ANP in evaluating Kanji complexity offers a replicable and rigorous framework for bridging the gap between traditional linguistic complexity measures and the real-world learning experience of non-native speakers. This study not only informs effective instructional practices but also opens avenues for adaptive learning systems and personalized Kanji curricula that respond to individual learner profiles and changing language needs.

## 4 Conclusion and future recommendations

This study proposes a transparent hybrid ANP–VIKOR methodological framework for evaluating the difficulty of learning Japanese Kanji using multiple linguistic criteria, expert judgments, and learner perceptions. The principal contribution of this research is the development of an interpretable decision-support framework for Kanji prioritization to inform educational planning and future research. The present study does not provide evidence of improved learning outcomes, psychological processes, or instructional effectiveness. Instead, it establishes a methodological basis that can be evaluated through future classroom-based and longitudinal studies.

The analysis of five high-frequency Kanji characters revealed that Kanji 3 (人) consistently emerged as the least complex across all scenarios. At the same time, Kanji 2 (学) was identified as the most complex, mainly due to its higher stroke count and lower semantic transparency. The sensitivity analysis confirmed the robustness of these rankings, with minor fluctuations in *Q* values across varying decision-maker emphasis scenarios (*v* = 0.3, 0.5, 0.7), but no significant changes in the overall order. This highlights the hybrid model’s reliability in identifying key complexity challenges faced by non-native learners.

The proposed ANP–VIKOR framework provides a transparent and systematic decision-support approach for evaluating the difficulty of learning Japanese Kanji by integrating expert knowledge, learner perceptions, and multiple linguistic criteria. The resulting rankings provide an objective basis for prioritizing Kanji and may assist researchers and curriculum developers in designing evidence-informed learning sequences. However, the present validation is based primarily on learner perception data and expert evaluations rather than on observed classroom learning outcomes. Therefore, the framework should be interpreted as a decision-support tool rather than as a validated instructional model. Its educational effectiveness and potential impact on curriculum design remain to be established through future classroom-based studies involving learning performance and longitudinal evaluation.

The findings underscore the practical significance of this approach for Japanese language educators and curriculum designers. By aligning instructional materials with data-driven complexity rankings, teachers can ensure a scaffolded learning experience, starting with less complex Kanji to build confidence and progressively introducing more challenging characters. Furthermore, integrating quantitative data (such as frequency and stroke count) and qualitative perceptions (expert and learner surveys) bridges the gap between traditional linguistic measures and the real-world difficulties language learners face.

Future research should further examine the robustness and generalizability of the proposed ANP–VIKOR framework through additional validation strategies. These may include ANP weight perturbation, leave-one-expert-out analysis, bootstrapping of learner ratings, alternative normalization methods, comparisons with other multi-criteria decision-making techniques such as TOPSIS and PROMETHEE, and evaluations involving independent expert panels. Furthermore, classroom-based implementation studies are needed to investigate the effectiveness of the proposed ranking framework in supporting Kanji learning, instructional sequencing, and curriculum development.

## Data Availability

The original contributions presented in the study are included in the article/Supplementary material, further inquiries can be directed to the corresponding author/s.

## References

[ref2] Halpern J. (2016). Compilation techniques for pedagogically effective bilingual learners’ dictionaries. Int. J. Lexicogr. 29, 323–338. doi: 10.1093/ijl/ecw023

[ref4] Inoue T. Georgiou G. K. Parrila R. (2022). Cross-script effects of cognitive-linguistic skills on Japanese hiragana and kanji: evidence from a longitudinal study. J. Cult. Cogn. Sci. 6, 119–134. doi: 10.1007/s41809-022-00099-8

[ref6] Kanno Y. (2008). Language and Education in Japan. Hampshire: Palgrave Macmillan.

[ref7] Konoeda K. (2024). ““My world has expanded”: Students’ counter-meta-standardization in language courses,” in Linguistic Counter-Standardization: Exploring Liberatory Language Practices around “Japanese”, (Berlin, Germany: De Gruyter Brill GmbH).

[ref8] Koyama N. (2022). “Academic Japanese: challenges and myths for learners of Japanese as a foreign language in the US,” in The Routledge Handbook of Asian Linguistics, (London, UK: Routledge), 650–666.

[ref9] Li X. Xiang J. Wu F.-X. Li M. (2021). A dual ranking algorithm based on the multiplex network for heterogeneous complex disease analysis. IEEE/ACM Trans. Comput. Biol. Bioinform. 19, 1993–2002. doi: 10.1109/TCBB.2021.3059046, 33577455

[ref10] Lu Y. Wang S. Chen R. Dui H. Gao J. Zhang C. (2026). Human-centric reliability prediction for IoT-enabled human-machine systems considering component hierarchical dependency. Int. J. Hum. Comput. Interact. 1, 1–26. doi: 10.1080/10447318.2026.2630080

[ref13] Mori Y. (2020). Perceptual differences about kanji instruction: native versus nonnative, and secondary versus postsecondary instructors of Japanese. Foreign Lang. Ann. 53, 550–575. doi: 10.1111/flan.12480

[ref14] Mori Y. Hasegawa A. Mori J. (2021). The trends and developments of L2 Japanese research in the 2010s. Lang. Teach. 54, 90–127. doi: 10.1017/S0261444820000336

[ref15] Otsuka S. Murai T. (2021). Cognitive underpinnings of multidimensional Japanese literacy and its impact on higher-level language skills. Sci. Rep. 11:2190. doi: 10.1038/s41598-021-81909-x, 33500509 PMC7838263

[ref16] Paxton S. Svetanant C. (2014). Tackling the kanji hurdle: investigation of kanji learning in non-kanji background learners. Int. J. Res. Stud. Lang. Learn. 3, 89–104. doi: 10.5861/ijrsll.2013.519

[ref17] Rasheed M. H. Khalid J. Ali A. Rasheed M. S. Ali A. (2024). Human resource analytics in the era of artificial intelligence: leveraging knowledge towards organizational success in Pakistan. J. Chin. Hum. Resour. Manag. 15, 3–20. doi: 10.47297/wspchrmWSP2040-800501.20241503

[ref18] Rodríguez Badas R.J. Kanji recognition with AI (2024)

[ref19] Rose H. (2017). The Japanese Writing system: Challenges, Strategies and Self-Regulation for Learning Kanji, Bristol, UK: Multilingual Matters vol. 116.

[ref20] Sandling E. The ito element: unravelling the thread in kanji. (2023)

[ref22] Shao S. Tian Y. Zhang Y. Zhang X. (2025). Knowledge learning-based dimensionality reduction for solving large-scale sparse multiobjective optimization problems. IEEE Transactions on Cybernetics 55, 3471–3484. doi: 10.1109/TCYB.2025.3558354, 40249685

[ref23] Tu Y. Huang C. Pan Y. Hwang G.-J. (2026). Mapping the structure of student burnout in online learning: an integrated Gaussian model and directed acyclic graph approach. Internet High. Educ. 70:101077. doi: 10.1016/j.iheduc.2026.101077

[ref24] Van A. N. T. Tran H. M. N. Kieu D. T. Trung L. T. Ngoc N. T. N. Cac T. T. T. (2024). Exploring work motivation and job effectiveness in Mekong delta’s hospitality: a study utilizing partial least squares structural equation modeling. J. Chin. Hum. Resour. Manag. 15, 101–112.

[ref25] Zhang R. Chen Y. (2026). What can multi-factors contribute to Chinese EFL learners’ implicit L2 knowledge? Int. Rev. Appl. Linguist. Lang. Teach. 64, 173–195. doi: 10.1515/iral-2024-0021

[ref26] Zheng W. (2024). Estimating word difficulty using stratified word familiarity. Cogent Arts Human. 11:2420467. doi: 10.1080/23311983.2024.2420467

[ref27] Zhu J. Chen Z. De Meo P. Guan J. Han Z. Shi W. (2026). KnowPath: an LLM-supported knowledge graph construction and path finding framework to explainable MOOC recommendations. ACM Trans. Inf. Syst. 44, 1–28. doi: 10.1145/3779436

