# Supplementary material for: A multi-criteria assessment of Japanese Kanji learning difficulty for educational prioritization using ANP–VIKOR
Source: Front Psychol. 2026 Aug 10;17:1902499. doi: 10.3389/fpsyg.2026.1902499 (PMC13500314; doi:10.3389/fpsyg.2026.1902499)
Supplement: Supplementary file 1 [file Table_2.DOCX]

***ANP Computational Procedure***

This appendix presents an example of the computational procedure used in the Analytic Network Process (ANP). The purpose is to demonstrate the sequence of calculations from expert judgments to the final criterion priorities. The values are provided to facilitate understanding of the methodology.

Table S1 presents the aggregated pairwise comparison matrix obtained by applying the geometric mean to the individual judgments of the ten experts. This aggregation method is widely adopted in ANP to derive a consensus comparison matrix.

**Table S1.** Pairwise comparison matrix representing the expert panel's aggregated judgments using Saaty's 1–9 scale.

| Criteria | Stroke Count | Frequency of Use | Semantic Transparency | Phonetic Variability |
| --- | --- | --- | --- | --- |
| Stroke Count | 1.000 | 2.000 | 2.000 | 3.000 |
| Frequency of Use | 0.500 | 1.000 | 1.000 | 2.000 |
| Semantic Transparency | 0.500 | 1.000 | 1.000 | 2.000 |
| Phonetic Variability | 0.333 | 0.500 | 0.500 | 1.000 |

**Table S2.** Normalized comparison matrix and corresponding priority vector.

| Criteria | Stroke Count | Frequency of Use | Semantic Transparency | Phonetic Variability | Priority |
| --- | --- | --- | --- | --- | --- |
| Stroke Count | 0.429 | 0.444 | 0.444 | 0.375 | 0.423 |
| Frequency of Use | 0.214 | 0.222 | 0.222 | 0.250 | 0.227 |
| Semantic Transparency | 0.214 | 0.222 | 0.222 | 0.250 | 0.227 |
| Phonetic Variability | 0.143 | 0.111 | 0.111 | 0.125 | 0.123 |

**Table S3.** Consistency assessment of the pairwise comparison matrix.

| Parameter | Value |
| --- | --- |
| Principal Eigenvalue (λmax) | 4.08 |
| Consistency Index (CI) | 0.027 |
| Random Index (RI) | 0.90 |
| Consistency Ratio (CR) | 0.030 |
| Decision | Acceptable (CR < 0.10) |

**Table S4.** Unweighted supermatrix showing the influence among the evaluation criteria.

| Criteria | Stroke Count | Frequency of Use | Semantic Transparency | Phonetic Variability |
| --- | --- | --- | --- | --- |
| Stroke Count | 0.42 | 0.40 | 0.38 | 0.35 |
| Frequency of Use | 0.23 | 0.25 | 0.24 | 0.22 |
| Semantic Transparency | 0.23 | 0.24 | 0.25 | 0.23 |
| Phonetic Variability | 0.12 | 0.11 | 0.13 | 0.20 |

**Table S5.** Weighted supermatrix after cluster weighting.

| Criteria | Stroke Count | Frequency of Use | Semantic Transparency | Phonetic Variability |
| --- | --- | --- | --- | --- |
| Stroke Count | 0.30 | 0.30 | 0.30 | 0.30 |
| Frequency of Use | 0.25 | 0.25 | 0.25 | 0.25 |
| Semantic Transparency | 0.25 | 0.25 | 0.25 | 0.25 |
| Phonetic Variability | 0.20 | 0.20 | 0.20 | 0.20 |

**Table S6.** limit supermatrix after convergence, yielding the criterion priorities used in the VIKOR analysis.

| Criterion | Final Weight |
| --- | --- |
| Stroke Count | 0.30 |
| Frequency of Use | 0.25 |
| Semantic Transparency | 0.25 |
| Phonetic Variability | 0.20 |
