# Supplementary material for: A multi-criteria assessment of Japanese Kanji learning difficulty for educational prioritization using ANP–VIKOR
Source: Front Psychol. 2026 Aug 10;17:1902499. doi: 10.3389/fpsyg.2026.1902499 (PMC13500314; doi:10.3389/fpsyg.2026.1902499)
Supplement: Supplementary file 2 [file Table_3.DOCX]

*Supplementary Appendix C*

Questionnaire for Evaluating the Learning Difficulty of Japanese Kanji Characters

Introduction

Dear Participant,

Thank you for participating in this study on Japanese Kanji learning. The purpose of this questionnaire is to evaluate learners' perceptions of Kanji learning difficulty based on four educational criteria.

Your responses will be used only for research purposes and will remain anonymous. There are no right or wrong answers. Please answer each question according to your own learning experience.

Estimated completion time: 15–20 minutes

Section A. Participant Information

Please select the most appropriate answer.

A1. Age

□ 18–30 years

□ 31–45 years

□ 46–60 years

□ Above 60 years

A2. Gender

□ Male

□ Female

□ Prefer not to say

A3. Country of Origin

A4. Native Language

A5. Highest Japanese Language Proficiency

□ JLPT N5

□ JLPT N4

□ JLPT N3

□ JLPT N2

□ JLPT N1

□ Not yet taken JLPT

A6. Years of Learning Japanese

□ Less than 1 year

□ 1–2 years

□ 3–5 years

□ More than 5 years

A7. Have you studied Japanese Kanji before?

□ Yes

□ No

Section B. Evaluation of Kanji Learning Difficulty

For each Kanji character, please rate the following four aspects using the scale below.

Rating Scale

| Score | Description |
| --- | --- |
| 1 | Very Easy |
| 2 | Easy |
| 3 | Moderate |
| 4 | Difficult |
| 5 | Very Difficult |

Kanji: 人

| Criterion | 1 | 2 | 3 | 4 | 5 |
| --- | --- | --- | --- | --- | --- |
| Stroke Count | □ | □ | □ | □ | □ |
| Frequency of Use | □ | □ | □ | □ | □ |
| Semantic Transparency | □ | □ | □ | □ | □ |
| Phonetic Variability | □ | □ | □ | □ | □ |

Evaluation Form

Repeat this table for every Kanji included in the study.

Kanji: __________

| Evaluation Criterion | 1 | 2 | 3 | 4 | 5 |
| --- | --- | --- | --- | --- | --- |
| 1. Stroke Count (Difficulty of writing this Kanji) | □ | □ | □ | □ | □ |
| 2. Frequency of Use (How frequently do you encounter this Kanji?) | □ | □ | □ | □ | □ |
| 3. Semantic Transparency (How easy is it to understand its meaning from its components?) | □ | □ | □ | □ | □ |
| 4. Phonetic Variability (Difficulty caused by multiple pronunciations) | □ | □ | □ | □ | □ |

*(Repeat for all selected Kanji characters.)*

Section C. Overall Opinion

C1.

Which factor makes Kanji learning most difficult?

□ Stroke Count

□ Frequency of Use

□ Semantic Transparency

□ Phonetic Variability

C2.

Which factor helps you learn Kanji most effectively?

□ Stroke Count

□ Frequency of Use

□ Semantic Transparency

□ Phonetic Variability

C3.

Do you think Kanji should be taught from the easier to the more difficult characters?

□ Strongly Disagree

□ Disagree

□ Neutral

□ Agree

□ Strongly Agree

C4.

Do you believe a data-driven ranking system could improve Kanji learning?

□ Strongly Disagree

□ Disagree

□ Neutral

□ Agree

□ Strongly Agree

Additional comments
